# Association Between Vitamin D Insufficiency and Impaired Bone Density Among Adolescents With Perinatally Acquired HIV Infection

**DOI:** 10.1093/ofid/ofae442

**Published:** 2024-09-19

**Authors:** Nyasha V Dzavakwa, Victoria Simms, Celia L Gregson, Molly Chisenga, Suzanne Filteau, Lackson Kasonka, Katharina Kranzer, Hildah Banda-Mabuda, Hilda Mujuru, Nicol Redzo, Cynthia Mukwasi-Kahari, Sarah L Rowland-Jones, Ulrich E Schaible, Rashida A Ferrand, VITALITY team, Emily Carr, Matthias Hauptmann, Grace McHugh, Ester Gea-Mallorqui, Christoph Leschczyk, Tafadzwa Madanhire, Tadious Manyanga, Tsitsi S Mudzingwa, Kudakwashe Mutasa, Cassandra Namukonda, N R Karen Sichibalo, Mizinga Tembo

**Affiliations:** The Health Research Unit Zimbabwe (THRU-Zim), Biomedical Research and Training Institute, Harare, Zimbabwe; MRC International Statistics and Epidemiology Group, London School of Hygiene and Tropical Medicine, London, United Kingdom; The Health Research Unit Zimbabwe (THRU-Zim), Biomedical Research and Training Institute, Harare, Zimbabwe; MRC International Statistics and Epidemiology Group, London School of Hygiene and Tropical Medicine, London, United Kingdom; The Health Research Unit Zimbabwe (THRU-Zim), Biomedical Research and Training Institute, Harare, Zimbabwe; Musculoskeletal Research Unit, Bristol Medical School, University of Bristol, Bristol, United Kingdom; University Teaching Hospital, Women and Newborn Hospital, Lusaka, Zambia; Department of Population Health, London School of Hygiene and Tropical Medicine, London, United Kingdom; University Teaching Hospital, Women and Newborn Hospital, Lusaka, Zambia; The Health Research Unit Zimbabwe (THRU-Zim), Biomedical Research and Training Institute, Harare, Zimbabwe; Division of Infectious Diseases and Tropical Medicine, LMU University Hospital, LMU Munich, Munich, Germany; Department of Clinical Research, London School of Hygiene and Tropical Medicine, London, United Kingdom; University Teaching Hospital, Women and Newborn Hospital, Lusaka, Zambia; Department of Paediatrics, University of Zimbabwe, Harare, Zimbabwe; The Health Research Unit Zimbabwe (THRU-Zim), Biomedical Research and Training Institute, Harare, Zimbabwe; The Health Research Unit Zimbabwe (THRU-Zim), Biomedical Research and Training Institute, Harare, Zimbabwe; MRC International Statistics and Epidemiology Group, London School of Hygiene and Tropical Medicine, London, United Kingdom; Nuffield Department of Medicine, University of Oxford, Oxford, United Kingdom; Cellular Microbiology, Priority Research Area Infections, Research Centre Borstel, Leibniz Lung Centre & Leibniz Research Alliance INFECTIONS, Borstel, Germany; Biochemical Microbiology & Immunochemistry, University of Lübeck, Lübeck, Germany; The Health Research Unit Zimbabwe (THRU-Zim), Biomedical Research and Training Institute, Harare, Zimbabwe; Department of Clinical Research, London School of Hygiene and Tropical Medicine, London, United Kingdom

**Keywords:** adolescent, bone density, HIV, musculoskeletal health, vitamin d

## Abstract

**Background:**

Stunting and pubertal delay are common among children growing up with human immunodeficiency virus (HIV) and are associated with bone and muscle impairments. We investigated factors associated with bone density and muscle function in adolescents living with HIV (ALWH).

**Methods:**

The VITALITY trial (PACTR202009897660297) investigated whether vitamin D and calcium supplementation improves musculoskeletal health among ALWH. A total of 842 ALWH aged 11–19 years, established on antiretroviral therapy (ART) for ≥6 months, were enrolled from HIV clinics in Zambia and Zimbabwe. Clinical history and examination were undertaken, and serum 25-hydroxyvitamin D_3_ (25[OH]D_3_) was measured. Dual-energy X-ray absorptiometry measured total-body-less-head bone mineral density adjusted for height (TBLH-BMD^HT^), and lumbar spine bone mineral apparent density (LS-BMAD) *z* scores. The association between a priori–defined covariates and musculoskeletal outcomes were investigated using baseline enrollment data and multivariable logistic regression.

**Results:**

TBLH-BMD^HT^  *z* scores were impaired (mean, −1.42 for male and −0.63 female participants), as were LS-BMAD *z* scores (mean −1.15 for male and −0.47 for female participants). In bivariate analysis, early pubertal stage, less physical activity, and older age at ART initiation were associated with lower TBLH-BMD^HT^  *z* scores. Younger age, early pubertal stage, and low socioeconomic status were associated with lower LS-BMAD *z* scores. Grip-strength-for-height and jump-power-for-height *z* scores were associated with lower TBLH-BMD^HT^ and LS-BMAD *z* scores. Low dietary vitamin D and calcium were associated with lower adjusted TBLH-BMD^HT^  *z* scores. Lower 25(OH)D_3_ was associated with lower adjusted TBLH-BMD^HT^ and LS-BMAD *z* scores.

**Conclusions:**

Deficits in bone density are common in ALWH. Vitamin D and calcium supplementation and promotion of exercise may improve musculoskeletal health among perinatally infected ALWH.

## Teaser

Adolescents with human immunodeficiency virus in urban Zimbabwe and Zambia have bone mineral density deficit, associated with low levels of both reported dietary vitamin D intake and measured serum 25-hydroxyvitamin D_3_. Supplementation may improve musculoskeletal health in this group.

**Figure 1.**
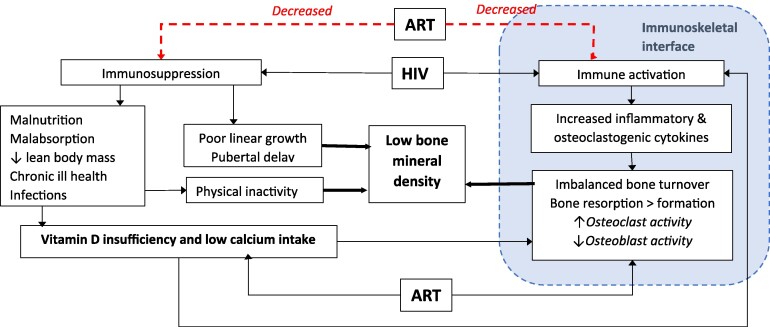
Risk factors for low bone density in children living with human immunodeficiency virus (HIV). Abbreviation: ART, antiretroviral therapy.

Human immunodeficiency virus (HIV) infection in children is associated with increased risk of multisystem comorbid conditions despite antiretroviral therapy (ART) [1, 2]. Perinatally acquired HIV has substantial adverse effects on growth, with resultant stunting and pubertal delay, as well as on musculoskeletal development and muscle function [3, 4]. These effects may be more common in low-income than in high-income settings, due to high rates of malnutrition and delayed ART initiation [5–7]. Studies in Africa have demonstrated reduced bone mass and strength in adolescents living with HIV (ALWH) [4, 7–9]. Drugs such as tenofovir disoproxil fumarate (TDF) (part of the World Health Organization–recommended first-line ART regimen for adults and adolescent [10]) may further impair bone accrual (Figure 1) [11].

Puberty is a crucial period for muscle and bone development; even after cessation of linear growth, mineral consolidation continues until peak bone mass (PBM) is reached [12]. PBM provides a mineral reservoir for later life and is a key determinant of adult bone mineral density (BMD), accounting for 60% of lifetime osteoporosis risk [8, 13]. A 10% decrease in PBM doubles adult fracture risk [11, 14]. Thus, the “growth spurt” during adolescence provides a window of opportunity to intervene and optimize musculoskeletal health for the remaining life course. In this article, we aim to understand the factors associated with impaired bone density and muscle function and specifically to interrogate whether low dietary intake and low concentrations of measured vitamin D are associated with impaired bone density and muscle function.

## METHODS

### Study Design and Setting

This was a cross-sectional study using baseline data from a double-blind, individually randomized placebo-controlled randomized trial of high-dose weekly vitamin D and daily calcium carbonate supplementation (VITALITY; registration PACTR202009897660297) [15]. Participants were enrolled from HIV clinics at Sally Mugabe Children's Hospital, Harare, Zimbabwe, and the University Teaching Hospital (Women and Newborn Hospital), Lusaka, Zambia, from February to November 2021.

### Participant Selection

HIV clinic attendees aged 11–19 years with perinatally acquired HIV, taking ART for ≥6 months and resident in Harare or Lusaka, were recruited. Perinatally acquired HIV was accepted as the most likely source if the following conditions were met: self-report of no sexual debut or blood transfusion, a history of orphanhood due to maternal HIV disease and/or a sibling death due to HIV, and characteristic clinical features (stunting, history of recurrent minor infections [skin, upper respiratory tract] in childhood, and/or planar warts). The 11–19-year age range was chosen to capture the full range of pubertal skeletal growth [4]. Adolescents were excluded if they were unaware of their HIV status (if aged ≥13 years), a coresident sibling was already enrolled in the trial, they had current tuberculosis or were on tuberculosis treatment, were pregnant or breastfeeding, had clinical evidence of rickets or metabolic bone disease, or were assessed as likely to have severe difficulty adhering to the trial drug. The sample size was based on an effect size of half the difference in bone density between ALWH and HIV-negative adolescents in this setting, from a previous study [15].

### Study Procedures

A standardized baseline questionnaire capturing sociodemographic data, HIV and ART history, prior fractures, physical activity, diet, and nutrition was administered to participants [7, 13]. Physical activity was assessed using the International Physical Activity Questionnaire, which calculates total physical activity as multiples of the resting metabolic rate (MET) in MET-minutes per week [16].

Tanner pubertal staging, based on observation of testes size for male and breast size for female participants, was performed by trained clinic research staff, and 3 measurements each were taken for weight in kilograms and height in centimeters (with a SECA 213 stadiometer), with the mean recorded. Height measurements >2 cm from the mean were discarded. Pubertal delay was defined as breast development at Tanner stage I for female participants aged ≥13 and testes development at Tanner stage I for male participants aged ≥14 years. Upper limb muscle grip strength and lower limb jumping power were assessed by means of Jamar hydraulic hand-held dynamometers (Patterson Medical) and standing long jumps, respectively. The maximum of 6 grip strength tests (3 with each hand) was taken as grip strength, in kilograms. The maximum of 3 standing long jump distances was recorded as jump distance, in centimeters. Calcium and vitamin D dietary consumption were calculated from a 7-item diet questionnaire based on a dietary diversity and food frequency tool [17], which has been contextually adapted by incorporating locally eaten vitamin D rich foods (eg, kapenta fish; Supplement A) [4]. Dietary calcium consumption was categorized as very low (<150 mg/d), low (150–299 mg/d), and moderate (≥300 mg/d), while dietary vitamin D was categorized as very low (<4 μg/d), low (4–5.9 μg/d), or moderate (≥6 μg/d). The British Nutrition Foundation reference nutrient intake in this age group is ≥1000 mg/d calcium for males (800 mg/d for females) and ≥10 μg/d vitamin D [18].

### Dual-Energy X-Ray Absorptiometry

Dual-energy x-ray absorptiometry (DXA) scans of the total body and lumbar spine (L1–L4) were performed by trained radiographers using a Hologic QDR Wi densitometer (Hologic) with Apex software (version 4.5) in Zambia and a Lunar iDXA (GE Healthcare) densitometer with ENCORE software (version 18) in Zimbabwe. Daily calibrations were conducted using the manufacturer-provided spine phantoms. DXA scans were repeated in 60 participants per country to determine reproducibility of measurements (Supplementary Table 1). A further cross-calibration phantom was scanned on both DXA machines every 6 months throughout data collection and confirmed no drift in measurement over time. DXA was also used to measure fat and lean muscle mass. An important limitation of DXA use in children is that 2-dimensional (areal) bone density values are highly dependent on body, and hence bone, size so that DXA underestimates bone density in small children [19]. Therefore size adjustment was used, specifically (1) total-body-less-head (TBLH) BMD adjusted for height (TBLH-BMD^HT^) and (2) lumbar spine bone mineral apparent density (LS-BMAD), calculated using the Carter method [20]. Sex and age-matched *z* scores were then generated using UK population reference data [21], as recommended by International Society for Clinical Densitometry (ISCD) guidelines, in the absence of local reference data [22]. Low TBLH-BMD^HT^ and LS-BMAD were defined as *z* score below −2.0 [21].

### Blood Tests

Finger-prick sample was collected for CD4 cell counts and were analyzed using Abbott CD4 PIMA analyzer. HIV viral load (VL) testing was done using the Qiagen rotor gene Q in Zambia and the Roche COBAS Ampliprep/COBAS Taqman48 in Zimbabwe. A small number of samples in Zambia were analyzed using the Aquois CL flow cytometer (Beckman Coulter) for CD4 cell count and Hologic Panther or GeneXpert for VL. HIV viral suppression was defined as a VL <60 copies/mL, based on the laboratory lower limit cutoff for viral detection across the 2 sites. Blood was also collected for plasma markers of bone metabolism, including 25-hydroxyvitamin D_3_ (25(OH)D_3_) concentrations, which were measured using Tandem mass spectrometry at the University of East Anglia. The 25(OH)D_3_ concentrations were categorized into <50, 50–75, and >75 nmol/L, based on a systematic review [23] and evidence that concentrations <75 nmol/L are associated with the lowest parathyroid hormone levels in this population (Madanhire et al, unpublished data).

### Data Management and Statistical Analysis

Data were collected onto tablets (Google Nexus; Google) with OpenDataKit software [24] and uploaded into Microsoft Access (Microsoft) for management. DXA data containing absolute values for bone mineral content and bone area were exported and linked to the main data set using anonymized participant study identification numbers. Analysis was performed using Stata software, version 17.0 (StataCorp).

Socioeconomic status (SES) was quantified as a continuous variable and divided into quintiles, based on measurements of household asset ownership, water source, and toilet type and using factor analysis. Height-for-age, body mass index (BMI)–for-age, sitting-height-for-age, grip-strength-for-height, and jump-power-for-height *z* scores were calculated. The UK reference standards were used to calculate height-for-age and grip strength-for-height *z* scores [25], to maintain consistency with the TBLH-BMD^HT^ and LS-BMAD *z* scores. Low grip-strength-for-height *z* scores were defined as a scores <0 because the distribution of scores was high. An internal jump-power-for-height *z* score by sex was calculated because there was no external reference population, with low jump-power-for-height *z* scores defined as those below −2. To adjust the TBLH-BMD *z* score for height, a regression equation of TBLH-BMD on height-for-age *z* score was used to calculate each child's predicted TBLH-BMD at a height-for-age *z* score of 0, and then *z* scores were derived from this predicted value.

To assess the association between age and SES quintiles, χ^2^ tests were used. Linear regression, adjusting for country as a random effect, was used to identify variables associated with TBLH-BMD^HT^, LS-BMAD, grip-strength-for-height, and jump-power-for-height *z* scores. Multivariate models were used to estimate the associations between the exposures, dietary vitamin D and calcium, and the outcomes, TBLH-BMD^HT^ and LS-BMAD *z* scores, adjusting for potential confounders and for country as a random effect. Potential confounders were identified by assessment of their association with the exposure and outcome.

The relationship between reported dietary vitamin D/calcium consumption and 25(OH)D_3_ category was assessed using χ^2^ tests, and the continuous data were presented in scatterplots. The mean and SD of the 4 outcomes TBLH-BMD^HT^, LS-BMAD, grip-strength-for-height, and jump-power-for-height *z* scores were reported by 25(OH)D_3_ category. Mixed-effects generalized linear models with binomial family and logit link were used to model the associations between TBLH-BMD^HT^, LS-BMAD, grip-strength-for-height and jump-power-for-height *z* scores with 25(OH)D_3_ category, with a random effect for country and fixed effects for sex and TBLH fat mass, because vitamin D is fat soluble and fat distribution varies by sex [26].

### Patient Consent Statement

Written informed consent was obtained from guardians, with written assent from participants aged <18 years. Participants aged ≥18 years provided independent consent. The study was approved by the Biomedical Research and Training Institute Institutional Review Board (AP158/2020), the Medical Research Council of Zimbabwe (A/2626), and the ethics committees of Harare Central Hospital (HCHEC 030320/12), London School of Hygiene and Tropical Medicine (22030), and the University of Zambia (1116-2020).

## RESULTS

### Participant Characteristics

In total 1432 attendees were screened, 860 screened eligible and 845 consented. Three were identified as ineligible after second-stage screening but none screened positive for rickets or a known metabolic bone disease (Supplementary Figure 1). Thus, 842 individuals aged 11–19 years were enrolled, 448 (53.2%) female and 420 (49.9%) from Zambia (Table 1 and Supplementary Table 2). The mean (SD) age was 15 (2.6) years, and 57.7% were in Tanner stage IV or V. Overall 84.7% of the total sample, and 96.4% of those aged <17 years, were enrolled in school.

**Table 1.** Descriptive Characteristics of the Study Population by Sex

Characteristic	Category	Participants, No. (%)^a^		
		Male (n = 394)	Female (n = 448)	Total (n = 842)
Country	Zimbabwe	204 (51.8)	218 (48.7)	422 (50.1)
	Zambia	190 (48.2)	230 (51.3)	420 (49.9)
Clinical characteristics				
Age, mean (SD), y		15.4 (2.8)	15.3 (2.8)	15.0 (2.6)
Tanner stage	I	34 (8.7)	43 (9.6)	77 (9.2)
	II	84 (21.4)	45 (10.1)	129 (15.4)
	III	90 (22.9)	76 (17.0)	166 (19.8)
	IV	94 (23.9)	113 (25.3)	207 (24.6)
	V	91 (23.2)	170 (38.0)	261 (31.1)
Pubertal delay	Yes	2/273 (0.7)	2/341 (0.6)	4/614 (0.7)
SES quintile	Highest	89 (22.6)	81 (18.1)	170 (20.2)
	4	76 (19.3)	91 (20.3)	167 (19.8)
	3	70 (17.8)	105 (23.4)	175 (20.8)
	2	82 (20.8)	80 (17.9)	162 (19.2)
	Lowest	77 (19.5)	91 (20.3)	168 (20.0)
Orphanhood	Yes (≥1 parent dead)	205 (52.0)	227 (50.7)	432 (51.3)
School registration	In school	326 (82.7)	387 (86.4)	713 (84.7)
Ever broken a bone	Yes	32 (8.1)	18 (4.0)	50/842 (5.9)
Physical activity	Low (<600 MET min/wk)	80 (20.3)	111 (24.8)	191 (22.7)
	Moderate (600–3000 MET min/wk)	224 (56.9)	246 (55.0)	470 (55.9)
	High (>3000 min/wk)	90 (22.8)	90 (20.1)	180 (21.4)
Daily dietary calcium consumption	Very low (<150 mg)	305 (77.4)	334 (74.6)	639 (75.9)
	Low (150–299 mg)	57 (14.5)	72 (16.1)	129 (15.3)
	Moderate (≥300 mg)	32 (8.1)	42 (9.4)	74 (8.8)
Daily dietary vitamin D consumption	Very low (<4 μg)	86 (21.8)	87 (19.4)	173 (20.6)
	Low 4 (<6 μg)	228 (57.9)	268 (59.8)	496 (58.9)
	Moderate (≥6 μg)	80 (20.3)	93 (20.8)	173 (20.5)
HIV characteristics				
ART line	First line	335 (85.0)	403 (90.2)	738 (87.8)
	Second line	59 (15.0)	44 (9.8)	103 (12.3)
Taking TDF	Yes	320 (81.2)	368 (82.1)	688 (81.7%)
Taking cotrimoxazole	Yes	110 (27.9)	131 (29.2)	241 (28.6)
VL	≥60 copies/mL	83 (21.1)	81 (18.1)	164/840 (19.5)
CD4 cell count	<500/µL	170/33 (43.3)	148/444 (35.8)	310/837 (39.3)
Anthropometry^b^				
Height, mean (SD), cm		154.5 (12.7)	151.4 (9.5)	152.8 (11.2)
Height-for-age *z* score, mean (SD)		−1.68 (1.06)	−1.21 (1.05)	−1.43 (1.08)
Stunted (height-for-age *z* score <−2.0)		145/394 (36.8)	103/447 (23.0)	248/841 (29.5)
DXA bone measurements				
Mean (SD)				
TBLH-BMC, kg		1.218 (0.440)	1.210 (0.339)	1.214 (0.390)
TBLH-BMD, g/cm^2^		0.783 (0.126)	0.810 (0.115)	0.797 (0.121)
TBLH-BMD *z* score		−2.12 (1.07)	−1.21 (1.13)	−1.64 (1.19)
TBLH–BMD-for-height *z* score		−1.42 (0.88)	−0.63 (0.94)	−1.00 (0.99)
LS-BMC, g		35.67 (13.37)	39.75 (12.08)	37.84 (12.85)
LS-BMAD, g/cm^3^		0.224 (0.047)	0.261 (0.051)	0.244 (0.052)
LS-BMAD *z* score		−1.15 (1.12)	−0.47 (1.07)	−0.79 (1.14)
TBLH–BMD-for-height *z* score <−2.0		96 (24.4)	36 (8.1)	132 (18.7)
LS-BMAD *z* score <−2.0		80 (20.3)	33 (7.4)	113 (13.5)
Muscle and fat measurements				
Mean (SD)				
TBLH lean mass, kg		30.8 (8.4)	28.0 (5.6)	29.3 (7.2)
TBLH fat mass, kg		6.5 (2.2)	12.1 (5.4)	9.5 (5.1)
Grip strength, kg		31.7 (12.0)	26.4 (8.0)	28.9 (10.4)
Grip-strength-for-height *z* score		2.34 (1.19)	2.43 (1.17)	2.39 (1.18)
Crude jump power, cm		157.8 (30.7)	136.0 (26.7)	146.2 (30.6)
Grip-strength-for-height *z* score <0		1/393 (0.3)	7/446 (1.6)	8/839 (1.0)
Jump-power-for-height *z* score <−2.0		43/392 (11.0)	27/444 (6.1)	70/836 (8.4)

Abbreviations: ART, antiretroviral therapy; BMAD, bone mineral apparent density; BMC, bone mineral content; BMD, bone mineral density; DXA, dual-energy x-ray absorptiometry; HIV, human immunodeficiency virus; LS, lumbar spine; SES, socioeconomic status; TBLH, total-body-less-head; TDF, tenofovir disoproxil fumarate; VL, viral load.

^a^Data represent no. (%) of participants unless otherwise specified.

^b^Anthropometry *z* scores were calculated using UK reference standards.

Most participants were taking a first-line ART regimen (with 81.7% taking TDF), and 103 (12.2%) a second-line regimen. Only 241 participants (28.6%) were taking cotrimoxazole prophylaxis, mainly in Zimbabwe. The median age at ART initiation was 5 years (interquartile range, 2–9 years). Lower SES was associated with younger age at ART initiation; in the most deprived quintile, 45.3% of adolescents had started ART before age 4 years and 20.0% at age ≥9 years, versus 32.1% and 31.0%, respectively, in the least deprived quintile (χ^2^ = 15.4; *P* = .05). The prevalence of virological suppression (defined as VL <60 copies/mL) was 80.5%.

#### Dietary Calcium and Vitamin D Consumption

Self-reported dietary calcium and vitamin D consumptions were low, with two-thirds of participants consuming <150 mg of calcium per day (Table 1). Lower dietary calcium and vitamin D consumptions were strongly associated with lower SES quintile (Supplementary Table 3). There was weak evidence of an association between 25(OH)D_3_ and categorical dietary vitamin D consumption (χ^2^ = 9.5; *P* = .052) (Supplementary Table 4).

### Associations With Skeletal Health

Overall, 29.5% of adolescents were stunted (height-for-age *z* score <−2), 36.8% of male and 23.0% of female participants. The mean TBLH-BMD *z* score (SD) was −1.64 (1.19). Notably after adjustment for height, the mean TBLH-BMD^HT^  *z* score (SD) increased to −1.00 (0.99). The mean LS-BMAD *z* score (SD) was −0.79 (1.14). Low TBLH-BMD^HT^ (*z* score <−2) was common, affecting 24% of male and 8% of female participants; similarly, low LS-BMAD was seen in 20% of male and 7% of female participants. Prior fracture was twice as frequent in male as in female participants (8.1% vs 4.0%, respectively) (Table 1).

**Figure 2.**
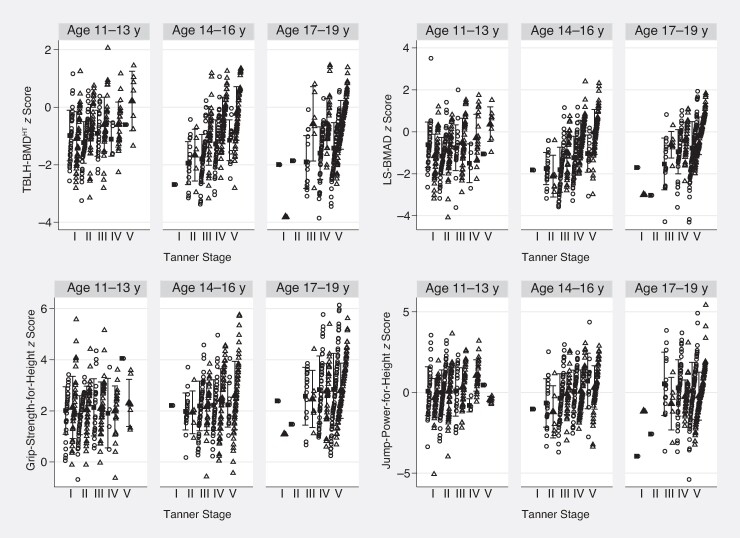
Mean and SD of total-body-less-head bone mineral density adjusted for height (TBLH-BMD^HT^), lumbar spine bone mineral apparent density (LS-BMAD), grip-strength-for-height, and jump-power-for-height *z* scores by age, sex, and Tanner stage.

In early Tanner stages the TBLH-BMD^HT^  *z* score was similar for male and female participants, while at later Tanner stages, female participants had greater TBLH-BMD^HT^ than male participants (Figure 2). LS-BMAD *z* score was higher in those who were older and in later Tanner stages (Figure 2).

In univariate analysis, lower TBLH-BMD^HT^  *z* score was associated with male sex, early Tanner stage, lower levels of physical activity, lower dietary intake of calcium and vitamin D, and older age at ART initiation (Table 2). In univariate analysis, lower LS-BMAD *z* score was associated with male sex, early Tanner stage, age <17 years, and lower SES, with consistent but weak evidence to support an association with low dietary calcium intake (Table 2).

**Table 2.** Univariate Analysis of Factors Associated With Total-Body-Less-Head Bone Mineral Density Adjusted for Height and Lumbar Spine Bone Mineral Apparent Density *z* Scores

Variable	Category	TBLH-BMD^HT^ *z* Score					LS-BMAD *z* Score				
		No.	Mean *z* Score	Country-Adjusted β Coefficient (95% CI)^a^	*P* Value	*R* ^2^ Value	No.	Mean *z* score	Country-Adjusted β Coefficient (95% CI)^a^	*P* Value	*R* ^2^ Value
Age	11–13 y	272	−0.90	Ref	.12	0.005	271	−0.82	Ref	.003	0.014
	14–16 y	288	−1.00	−0.11 (−.27 to .06)			288	−0.93	−0.10 (−.29 to .08)		
	17–19 y	281	−1.08	−0.17 (−.34 to –.01)			281	−0.61	0.21 (.02 to .41)		
Sex	Male	394	−1.42	Ref	<.001	0.158	394	−1.15	Ref	<.001	0.088
	Female	447	−0.63	0.79 (.67 to .91)			446	−0.47	0.68 (.53 to .83)		
Pubertal status	Tanner I	77	−1.11	Ref	<.001	0.036	77	−0.96	Ref	<.001	0.089
	Tanner II	129	−1.21	−0.10 (−.38 to .17)			129	−1.21	−0.25 (−.56 to .06)		
	Tanner III	166	−1.22	−0.11 (−.37 to .15)			166	−1.15	−0.19 (−.49 to .11)		
	Tanner IV	208	−0.93	0.18 (−.07 to .44)			207	−0.70	0.26 (.02–55)		
	Tanner V	261	−0.77	0.34 (.09–59)			261	−0.36	0.61 (.33–88)		
SES quintile	Highest	170	−1.06	Ref	.11	0.009	167	−0.60	Ref	.007	0.017
	4	166	−1.14	−0.19 (−.40 to .02)			162	−0.65	−0.05 (−.29 to .20)		
	3	175	−0.95	−0.27 (−.48 to .06)			174	−0.77	−0.16 (−.41 to .08)		
	2	162	−0.98	−0.08 (−.29 to .13)			167	−0.91	−0.31 (−.56 to –.07)		
	Lowest	168	−0.87	−0.11 (−.32 to .10)			170	−0.99	−0.39 (−.63 to –.14)		
Orphanhood	No	409	−1.00	Ref	.83	0.0001	409	−0.80	Ref	.67	0.0002
	Yes (≥1 parent dead)	432	−0.99	0.01 (−.12 to .15)			431	−0.77	0.03 (−.12 to .19)		
Physical activity level	Low (<600 MET min/wk)	191	−1.14	−0.30 (−.50 to –.10)	.015	0.010	190	−0.76	−0.11 (−.34 to .12)	.14	0.005
	Moderate (600–3000 MET min/wk)	469	−1.00	−0.16 (−.33 to .01)			469	−0.85	−0.20 (−.39 to –.00)		
	High (>3000 MET min/wk)	180	−0.84	Ref			180	−0.65	Ref		
Daily dietary calcium	Moderate (≥300 mg)	74	−0.75	Ref	.030	0.008	74	−0.52	Ref	.08	0.006
	Low (150–299 mg)	129	−0.90	−0.15 (−.43 to .13)			129	−0.73	−0.22 (−.53 to .11)		
	Very low (<150 mg)	638	−1.04	−0.29 (−.53 to –.05)			637	−0.83	−0.31 (−.58 to –.03)		
Daily dietary vitamin D	Moderate (≥6.0 μg)	173	−1.17	Ref	.03	0.009	172	−0.72	Ref	.63	0.001
	Low (4.0–5.9 μg)	496	−0.96	−0.05 (−.22 to .12)			495	−0.79	−0.08 (−.27 to .12)		
	Very low (<4.0 μg)	172	−0.92	−0.26 (−.47 to –.05)			173	−0.83	−0.12 (−.36 to .13)		
HIV VL	<60 Copies/mL	675	−0.98	Ref	.27	0.002	675	−0.78	Ref	.97	0.0000
	≥60 Copies/mL	164	−1.07	−0.10 (−.27 to .07)			163	−0.79	−0.00 (−.20 to .19)		
Age at ART initiation	<4 y	300	−0.86	Ref	.006	0.012	299	−0.78	Ref	.92	0.0002
	4–8 y	320	−1.03	−0.17 (−.32 to .01)			321	−0.77	0.01 (−.17 to .19)		
	>8 y	221	−1.13	−0.27 (−.44 to –.10)			220	−0.81	0.03 (−.23 to .17)		
Taking TDF	No	154	−0.96	Ref	.57	0.0004	154	−0.71	Ref	.31	0.001
	Yes	687	−1.01	−0.05 (−.22 to .12)			686	−0.80	−0.10 (−.30 to .10)		

Abbreviations: ART, antiretroviral therapy; CI, confidence interval; HIV, human immunodeficiency virus; LS-BMAD, lumbar spine bone mineral apparent density; MET, metabolic equivalent of task; Ref, reference standard; SES, socioeconomic status; TBLH-BMD^HT^, total-body-less-head bone mineral density adjusted for height; TDF, tenofovir disoproxil fumarate; VL, viral load.

^a^Country-adjusted β coefficient represents the difference in *z* score between a given category and the reference category, modelled with a random intercept for country.

After adjustment for sex and Tanner stage as fixed effects, and country as a random effect, each 10-μg/d increase in intake of vitamin D was associated with a 0.70 (95% confidence interval [CI], .23–1.16; *P* = .003) higher TBLH-BMD^HT^  *z* score, and each dietary calcium intake increase of 100 mg/d was associated with a 0.12 (.05–0.19; *P* < .001) higher TBLH-BMD^HT^  *z* score.

### Associations With Muscle Function

As expected, male participants had higher grip strength than female participants (mean [SD], 32 [12] vs 26 [8] kg). However, after accounting for height, there was little difference in the grip-strength-for-height *z* scores, which were high in both sexes (Table 1). Older age, later Tanner stage, and greater levels of physical activity were all associated with greater grip-strength-for-height *z* scores, whereas dietary calcium and vitamin D were not (Table 3 and Figure 2). Both TBLH-BMD^HT^ and LS-BMAD *z* scores were associated with higher grip-strength-for-height *z* scores (Table 3). After adjustment for age and Tanner stage, an increase of 1000 MET min/wk in physical activity was associated with an increase in grip-for-height *z* score of 0.025 (95% CI, .004–.06; *P* = .02).

**Table 3.** Univariate Analysis of Factors Associated With Grip-Strength-for-Height and Jump-Power-for-Height *z* Scores

Variable	Category	Grip-Strength-for-Height *z* Score					Jump-Power-for-Height *z* Score				
		No.	Mean *z* Score	Country-Adjusted β Coefficient (95% CI)^a^	*P* Value	*R* ^2^ Value	No.	Mean *z* Score	Country-Adjusted β Coefficient (95% CI)^a^	*P* Value	*R* ^2^ Value
Age	11–13 y	270	2.05	Ref	<.001	0.072	271	0.09	Ref	.50	0.002
	14–16 y	288	2.31	0.29 (.10–.47)			287	−0.03	−0.11 (−.36 to .13)		
	17–19 y	281	2.78	0.75 (.57–.93)			279	−0.05	−0.14 (−39 to .11)		
Sex	Male	393	2.33	Ref	.23	0.002	392	0.00	Ref	.97	0.000
	Female	446	2.42	0.09 (−.06 to .25)			445	0.00	−0.00 (−.20 to .20)		
Pubertal status	Tanner I	75	2.07	Ref	<.001	0.066	76	−0.23	Ref	.26	0.006
	Tanner II	129	1.92	−0.16 (−.47 to .16)			129	−0.14	0.09 (−.33 to .51)		
	Tanner III	166	2.20	0.13 (−.18 to .43)			165	−0.02	0.20 (−.20 to .60)		
	Tanner IV	208	2.47	0.39 (.10–68)			207	0.15	0.38 (−.01 to .76)		
	Tanner V	261	2.73	0.65 (.37–93)			260	0.03	0.26 (−.12 to .64)		
SES (quintile)	Highest	168	2.53	Ref	.15	0.0081	166	−0.00	Ref	.93	0.001
	4	162	2.38	−0.16 (−.40 to .09)			161	0.05	0.06 (−.25 to .38)		
	3	175	2.40	−0.14 (−.38 to .10)			175	0.05	−0.04 (−.36 to .28)		
	2	165	2.36	−0.18 (−.42 to .07)			166	−0.04	0.05 (−.26 to .37)		
	Lowest	168	2.21	−0.32 (−.57 to –.08)			169	0.06	−0.06 (−.38 to .26)		
Orphanhood	No	408	2.37	Ref	.90	0.0000	408	0.05	Ref	.36	0.001
	Yes (≥1 parent dead)	431	2.38	0.01 (−.14 to .17)			428	−0.04	−0.09 (−.29 to .11)		
Physical activity level	Low (<600 MET min/wk)	191	2.02	−0.63 (−.86 to –.40)	<.001	0.035	190	0.32	0.41 (.11–71)	.003	0.014
	Moderate (600–3000 MET min/wk)	467	2.41	−0.24 (−.43 to –.04)			466	−0.09	−0.01 (−.26 to .24)		
	High (>3000 MET min/wk)	180	2.65	Ref			180	−0.08	Ref		
Daily dietary calcium	Moderate (≥300 mg)	74	2.36	Ref	.98	0.0001	72	−0.00	Ref	.56	0.001
	Low (150–299 mg)	129	2.36	0.01 (−0.32 to .33)			128	−0.12	−0.12 (−0.55 to .30)		
	Very low (<150 mg)	636	2.38	0.02 (−0.25 to .30)			636	0.03	0.03 (−0.33 to .39)		
Daily dietary vitamin D	Moderate(≥6.0 μg)	173	2.42	Ref	.16	0.0043	170	−0.28	Ref	.017	0.010
	Low (4.0–5.9 μg)	494	2.41	0.00 (−0.20 to .19)			495	0.06	0.33 (.08–59)		
	Very low (<4.0 μg)	172	2.23	−0.19 (−.43 to .05)			171	0.13	0.40 (.09–72)		
HIV VL	<60 Copies/mL	674	2.35	Ref	.25	0.0016	671	0.07	Ref	.011	0.008
	≥60 Copies/mL	163	2.47	0.12 (−0.08 to .31)			163	−0.26	−0.33 (−0.58 to –.07)		
Age at ART initiation	<4 y	299	2.31	Ref	.52	0.0016	298	0.03	Ref	.75	0.0007
	4–8 y	320	2.41	0.09 (−0.09 to .28)			321	−0.05	−0.08 (−0.31 to .15)		
	>8 y	220	2.41	0.09 (−0.11 to .29)			218	0.03	−0.00 (−0.26 to .25)		
Taking TDF	No	153	2.44	Ref	.41	0.0008	154	−0.10	Ref	.33	0.001
	Yes	686	2.36	−0.08 (−0.28 to .12)			682	0.03	0.13 (−0.13 to .39)		
TBLH-BMD^HT^ *z* score	≥−2	706	2.46	Ref	<.001	0.034	704	0.07	Ref	.001	0.012
	<−2	131	1.89	−0.58 (−0.78 to –.37)		…	131	−0.37	−0.45 (−0.72 to –.18)	…	…
LS-BMAD *z* score	≥−2	725	2.42	Ref	.008	0.157	721	0.08	Ref	<.001	0.017
	<−2	112	2.06	−0.29 (−0.50 to –.08)			113	−0.49	−0.57 (−0.86 to –.28)		

Abbreviations: ART, antiretroviral therapy; CI, confidence interval; HIV, human immunodeficiency virus; LS-BMAD, lumbar spine bone mineral apparent density; MET, metabolic equivalent of task; Ref, reference standard; SES, socioeconomic status; TDF, tenofovir disoproxil fumarate; TBLH-BMD^HT^, total-body-less-head bone mineral density adjusted for height; VL, viral load.

^a^Country-adjusted β coefficient represents the difference in *z* score between a given category and the reference category, modelled with a random intercept for country.

Mean jump-power-for-height *z* scores increased with pubertal stage, up to age 16 years (Figure 2). Lower jump-power-for-height *z* scores were associated with low TBLH-BMD^HT^ and LS-BMAD *z* scores (Table 3). Decreased physical activity and decreased vitamin D dietary intake were both associated with higher jump-power-for-height *z* scores. After adjustment for age and Tanner stage, an increase in physical activity of 1000 MET min/wk was associated with a decrease in jump-power-for-height *z* score of 0.03 (95% CI, .06.00; *P*= .02). The association with physical activity category remained after adjusting for age and Tanner stage (ß = 0.19 [95% CI, .34–.42]; *P* = .01). HIV viral suppression was strongly associated with higher jump-power-for-height *z* scores, but there was no association between VL and grip-strength-for-height *z* scores (Table 3).

### Association of 25(OH)D_3_ With Skeletal Health and Muscle Function

After adjustment for sex and TBLH fat mass, lower 25(OH)D_3_ levels were associated with increased risk of low TBLH-BMD^HT^ and LS-BMAD *z* scores (Table 4). Specifically, adolescents with 25(OH)D_3_ <50 nmol/L had more than twice the risk of low TBLH-BMD^HT^ and LS-BMAD *z* scores than those with 25(OH)D_3_ > 75 nmol/L. No association was seen between 25(OH)D_3_ and low grip-strength-for-height or jump-power-for-height *z* scores.

**Table 4.** Association of 25-Hydroxyvitamin D_3_ Concentrations With Bone Density and Muscle Function Outcomes^a^

25(OH)D_3_	No.	TBLH-BMD^HT^ *z* Score <−2			LS-BMAD *z* Score <−2			Grip-Strength-for-Height *z* Score <0			Jump-Power-for-Height *z* Score <−2		
		RR (95% CI)	*P* Value		RR (95% CI)	*P* Value		RR (95% CI)	*P* Value		RR (95% CI)	*P* Value	
			1	2		1	2		1	2		1	2
>75 nmol/L	203	Ref	…		Ref	…		-^b^	…	…	Ref	…	
50–75 nmol/L	520	1.23 (.72–2.10)	.44	.013	1.34 (.80–2.24)	.27	.047	Ref	…	…	1.24 (.70–2.21)	.47	.73
<50 nmol/L	119	2.39 (1.25–4.57)	.009		2.24 (1.17–4.28)	.02		1.58 (.31–8.05)	.58	…	1.01 (.37–2.72)	.99	

Abbreviations: 25(OH)D_3_, 25-hydroxyvitamin D_3_; CI, confidence interval; LS-BMAD, lumbar spine bone mineral apparent density; Ref, reference standard; RR, risk ratio; TBLH-BMD^HT^, total-body-less-head bone mineral density adjusted for height,

^a^Associations based on generalized linear mixed models adjusting for sex and TBLH fat mass (in kilograms) as fixed effects, with a random intercept and slope for country. *P* value 1 is derived from the model, and *P* value 2 from the Wald test.

^b^All participants with 25(OH)D>75nmol/L had a grip strength for height z score > 0 so this category was dropped from the model due to collinearity.

## DISCUSSION

This study showed a high prevalence of low bone density in ALWH, especially males, among whom nearly a quarter were affected. Factors associated with low bone density *z* score, at ≥1 skeletal sites, included being male, at early Tanner stage, older at the time of ART initiation, more socially deprived and relatively physically inactive, and consuming low levels of dietary calcium and vitamin D. By contrast, grip strength was well preserved with only 8 participants (1.0%) having a grip-strength-for-height *z* score <0. Those who were older, more pubertally mature, and most physically active had the greatest muscle strength. HIV viral suppression was associated with better muscle power, but surprisingly, participants who were more physically active and had higher dietary vitamin D intake had lower muscle power. Taken together, findings point toward a number of potentially modifiable factors associated with musculoskeletal health.

A 30% prevalence of stunting is still commonly seen in ALWH in Southern Africa [27]; however, the prevalence of low size-adjusted bone density (ie, *z* score below −2) in this setting is much less frequently studied. That a quarter of male participants have low bone density, exceeds the estimates of the 2 previous studies in Zimbabwe. The first identified 12.5% and 14.6% prevalences of low TBLH-BMC^LBM^ (bone mineral content for lean mass adjusted for height) and LS-BMAD, respectively, in children living with HIV (mean age, 12.7 years) [7], while the second identified prevalences of 10% and 14%, respectively, in similarly aged children [4]. The finding of greater bone deficits in an older age group is a concerning pattern for future adulthood, given that a bone density *z* score below −2 approximates to a 4-fold increase in fracture risk, compared with a *z* score of 0 [28]. No association was detected between TDF use and bone density in this study, possibly because >80% of participants were taking TDF, and because we did not collect data on duration of use or whether those not taking TDF had ever taken it in the past.

Male adolescents had lower bone density than female participants. This is likely to be due to greater delays in puberty among males. Growth of the skeleton during puberty consists of rapid linear growth (corresponding to height increase) followed by a period of consolidation to mineralize the skeleton. In our study, male participants experienced linear growth at an age when the reference population were in the later consolidation phase, hence the lower bone density *z* score.

We found that 25(OH)D_3_ level was associated with both TBLH-BMD^HT^ and LS-BMAD *z* scores. This finding is supported by a study of 412 ALWH in the United States, in which low 25(OH)D_3_ (defined as ≤50 nmol/L) was associated with 0.38 lower total-body BMD *z* scores, although there was no association with LS-BMD [29]. Another study in the United States of 101 males aged 15–22 years with HIV found that low 25(OH)D_3_ (<50 nmol/L) was associated with increased risk of “bone toxicity” (defined as no increase in bone density over 48 weeks for adolescents or a decrease in adults) [30]. Two small studies of adolescents in Brazil and Thailand found no association between 25(OH)D_3_ and bone density [31, 32], but when those in the Thai group were given vitamin D and calcium supplementation their LS bone density increased [33].

The calcium intake reported by participants in the current study falls well below UK recommended levels [18]. Recent evidence has suggested that populations habituated to low dietary calcium intake (as is common in the sub-Saharan Africa region [23]) may not benefit from normalizing calcium intakes to international standards [34]. The associations identified in our study between very low dietary calcium levels and bone density suggest otherwise and point to the need to evaluate calcium supplementation in this specific population of ALWH, who have high mineral demands during the “growth spurt,” when 20%–30% of total-body bone mineral is rapidly accrued toward PBM [35]. Low SES was associated with low dietary calcium intake; calcium-rich foods are few, expensive, and uncommonly eaten in the region [36], making dietary modification a challenge. Dietary calcium is often consumed as part of a protein-rich diet, which may independently benefit musculoskeletal growth [37]. The associations in this study between dietary vitamin D/calcium and bone density and between dietary vitamin D and 25(OH)D_3_ provide evidence for the validity of the 7-item dietary questionnaire, for further musculoskeletal studies in the region.

In contrast to the skeletal findings, participants had above-average muscle strength, when compared against the same UK reference population as was used to generate bone density *z* scores, suggesting that Southern African children have inherently better muscle function, and/or that HIV is characterized by specific skeletal deficits, while muscle function is relatively preserved. However, children living with HIV in Zimbabwe, particularly male adolescents in late-stage puberty, have previously been reported to have lower muscle strength and power than HIV-negative peers [3]. Our findings suggest that uncontrolled VL is associated with lower muscle power, consistent with chronic inflammation leading to intramuscular fat, which may impair muscle function [3, 38]. The fact that muscle strength was higher in those reporting more physical activity is promising when considering potential public health interventions to maximize musculoskeletal health.

The current study has several strengths. Following ISCD recommendations [21], we calculated size-adjusted bone density, an important approach in a population with high prevalence of stunting. The mean TBLH-BMD *z* score was −1.64, corrected to −1.00 after adjustment, demonstrating the risk of overestimating bone density deficits if ISCD guidance is not followed. We acknowledge several limitations. In the absence of published African reference data, bone density and grip strength *z* scores were calculated using UK reference data, which are unlikely to be directly comparable to study participants. The dietary intake questionnaire used crude categories, was measured only over a week, and was subject to reporting bias. In addition, most vitamin D is produced from sun exposure, which we were unable to measure. Nonetheless, we did find association between the survey results and harder outcomes. No external reference population data exist for jump-power-for-height *z* scores.

Furthermore, based on feedback from the study field team, while competitive adolescent boys enjoyed the challenge of jumping, body-conscious adolescent girls—who, for example, may not have been wearing a suitable bra—were less accepting. This may have led to underestimates of muscle power in later Tanner stage and might explain the unexpected negative associations of jump power with physical activity and dietary vitamin D. Had resources allowed, jumping mechanography would have been preferable [39]. Finally, the analysis was cross-sectional, and causality cannot be inferred.

In conclusion, the current study identified a high prevalence of low bone density in ALWH; those who were male, in early Tanner stage, older at ART initiation, more socially deprived, or physically inactive and those who consumed less dietary calcium and vitamin D were at greatest risk. While deficits in muscle strength were less common, greater physical activity and greater bone density were associated with better muscle strength. These findings highlight several modifiable factors associated with musculoskeletal health in ALWH, potentially amenable to intervention.

## Supplementary Data

Supplementary materials are available at *Open Forum Infectious Diseases* online. Consisting of data provided by the authors to benefit the reader, the posted materials are not copyedited and are the sole responsibility of the authors, so questions or comments should be addressed to the corresponding author.

## Supplementary Material

ofae442_Supplementary_Data

## Notes

***VITALITY team.*** VITALITY team members include Emily Carr, Matthias Hauptmann, Grace McHugh, Ester Gea-Mallorqui, Christoph Leschczyk, Tafadzwa Madanhire, Tadious Manyanga, Tsitsi S. Mudzingwa, Kudakwashe Mutasa, Cassandra Namukonda, N. R., Karen Sichibalo, and Mizinga Tembo.

***Disclaimer.*** The funders had no role in the study design, data collection and analysis, decision to publish, or preparation of the manuscript.

***Financial support*.** This work is supported by the European and Developing Countries Clinical Trials Partnership (grant RIA2018CO-2512 funding the VITALITY trial); the Wellcome Trust (senior fellowship 206316/Z/17/Z to R. A. F.); and joint funding under the Medical Research Council (United Kingdom)/Department for International Development (United Kingdom) Concordat agreement, supported by the European Union under the EDCTP2 program (reference MR/K012126/1; grant G0700837 to N. D. and V. S.).
