# Supplementary material for: Association Between Vitamin D Insufficiency and Impaired Bone Density Among Adolescents With Perinatally Acquired HIV Infection
Source: Open Forum Infect Dis. 2024 Sep 19;11(9):ofae442. doi: 10.1093/ofid/ofae442 (PMC11411771; doi:10.1093/ofid/ofae442)
Supplement: ofae442_Supplementary_Data [file ofae442_supplementary_data.zip › Vitality baseline supplementary tables.docx]

Supplementary Table S1: precision error of DXA measurements

| Country | N | Measurement | Root mean square standard deviation  (RMS-SD) | Root mean square percent coefficient of variation  (RMS-%CV) |
| --- | --- | --- | --- | --- |
| Zambia | 60 | Total body less head | 0.012 g/cm^2^ | 1.56% |
|  | 60 | Lumbar spine | 0.012 g/cm^2^ | 1.63% |
| Zimbabwe | 60 | Total body less head | 0.013 g/cm^2^ | 1.73% |
|  | 60 | Lumbar spine | 0.013 g/cm^2^ | 1.42% |

Supplementary Table S2: Descriptive characteristics of the study population, stratified by country

|  |  | Zimbabwe | Zambia | Total, n (%) |
| --- | --- | --- | --- | --- |
|  |  | 422 | 420 | 842 |
| Clinical characteristics |  |  |  |  |
| Age, mean (SD) | Years | 15.5 (2.6) | 15.0 (2.5) | 15.2 (2.6) |
| Tanner stage, n (%) | I | 46 (10.9) | 31 (7.4) | 77 (9.2) |
|  | II | 57 (13.5) | 72 (17.1) | 129 (15.4) |
|  | III | 76 (18.0) | 90 (21.4) | 166 (19.8) |
|  | IV | 106 (25.1) | 102 (24.3) | 208 (24.7) |
|  | V | 137 (32.5) | 125 (29.8) | 262 (31.1) |
| Pubertal delay, n (%) | Yes | 2/313 (0.6) | 2/301 (0.7) | 4/614 (0.7) |
| SES | Highest | 90 (21.3) | 80 (19.1) | 170 (20.2) |
|  | 4 | 81 (19.2) | 86 (20.5) | 167 (19.8) |
|  | 3 | 74 (17.5) | 101 (24.1) | 175 (20.8) |
|  | 2 | 85 (20.1) | 77 (18.3) | 162 (19.2) |
|  | Lowest | 92 (21.8) | 76 (18.1) | 168 (20.0) |
| Orphanhood, n (%) | One or both parents dead | 227 (53.8) | 205 (48.8) | 432 (51.3) |
| School registration, n (%) | In school | 340 (80.6) | 373 (88.8) | 713 (84.7) |
| Ever broken a bone, n (%) | Yes | 23 (5.5) | 27 (6.4) | 50 (5.9) |
| Physical activity, MET mins/week, n (%) | Low, <600 | 41 (9.7) | 150 (35.7) | 191 (22.7) |
|  | Moderate, 600-3000 | 260 (61.8) | 210 (50.0) | 470 (55.9) |
|  | High, >3000 | 120 (28.5) | 60 (14.3) | 180 (21.4) |
| Daily dietary calcium consumption, mg, n (%) | Very low, <150mg | 318 (75.4) | 321 (76.4) | 639 (75.9) |
|  | Low, 150-299mg | 63 (14.9) | 66 (15.7) | 129 (15.3) |
|  | Moderate ≥300mg | 41 (9.7) | 33 (7.9) | 74 (8.8) |
| Daily dietary vitamin D consumption, μg, n (%) | Very low <4μg | 51 (12.1) | 122 (29.1) | 173 (20.6) |
|  | Low, 4-<6μg | 261 (61.9) | 235 (56.0) | 496 (58.9) |
|  | Moderate ≥6μg | 110 (26.1) | 63 (15.1) | 173 (20.5) |
| HIV characteristics |  |  |  |  |
| ART line, n (%) | First line | 345 (81.8) | 393 (93.8) | 738 (87.8) |
|  | Second line | 77 (18.3) | 26 (6.2) | 103 (12.3) |
| Taking TDF, n (%) | Yes | 328 (77.7) | 360 (85.7) | 688 (81.7%) |
| Taking cotrimoxazole, n (%) | Yes | 238 (56.4) | 3 (0.7) | 241 (28.6) |
| Viral load, copies/ml, n (%) | ≥60 | 100/422 (23.7) | 64/418 (15.3) | 164/840 (19.5) |
| CD4 count, cells/mm^3^ n (%) | <500 | 143/420 (34.1) | 186/417 (44.6) | 329/837 (39.3) |
| Anthropometry |  |  |  |  |
| Height, cm | Mean (SD) | 153.6 (11.9) | 152.1 (10.6) | 152.9 (11.2) |
| Height for age Z-score | Mean (SD) | -1.46 (1.10) | -1.40 (1.06) | -1.43 (1.08) |
| Stunted  (Height for age Z-score <-2.0) | N (%) <-2.0 | 124/421 (29.5) | 124 (29.5) | 248/841 (29.5) |
| DXA Bone measurements |  |  |  |  |
| TBLH BMC (kg) | Mean (SD) | 1.396 (0.392) | 1.030 (0.287) | 1.214 (0.389) |
| TBLH BMD (g/cm^2^) | Mean (SD) | 0.830 (0.129) | 0.764 (0.102) | 0.797 (0.121) |
| TBLH BMD for Z-score | Mean (SD) | -1.32 (1.14) | -1.95 (1.15) | -1.64 (1.19) |
| TBLH BMD for height Z-score | Mean (SD) | -0.72 (0.95) | -1.27 (0.96) | -1.00 (0.99) |
| TBLH BMD for height Z-score | N (%) <-2.0 | 37 (8.8) | 95 (22.6) | 132 (15.7) |
| LS BMC (g) | Mean (SD) | 39.58 (13.77) | 36.10 (11.63) | 37.84 (12.85) |
| LS BMAD (g/cm^3^) | Mean (SD) | 0.275 (0.045) | 0.212 (0.039) | 0.244 (0.052) |
| LS-BMAD Z-score | Mean (SD) | -0.73 (1.07) | -0.84 (1.21) | -0.79 (1.14) |
| LS-BMAD Z-score | N (%) <-2.0 | 48 (11.4) | 65 (15.5) | 113 (13.5) |
| Muscle and fat measurements |  |  |  |  |
| TBLH lean mass, kg | Mean (SD) | 29.8 (7.5) | 28.8 (6.7) | 29.3 (7.2) |
| TBLH fat mass, kg | Mean (SD) | 9.8 (5.3) | 9.2 (4.7) | 9.5 (5.1) |
| Grip strength, kg | Mean (SD) | 31.9 (11.0) | 25.7 (8.6) | 28.8 (10.4) |
| Grip strength for height Z-score | Mean (SD) | 2.82 (1.12) | 1.93 (0.98) | 2.38 (1.14) |
| Grip strength-for-height Z-score | N (%) < 1 | 2/419 (0.5) | 6/420 (1.4) | 8/839 (1.0) |
| Crude jump power, cm | Mean (SD) | 136.3 (32.4) | 156.1 (25.2) | 146.2 (30.6) |
| Jump power-for-height Z-score | N (%) <-2.0 | 61/417 (14.6) | 9/419 (2.2) | 70/836 (8.4) |

ART: antiretroviral therapy. BMAD: bone mineral apparent density. BMC: bone mineral content. BMD: bone mineral density. LS: lumbar spine. SES: socioeconomic status TBLH: total body less head. TDF: tenofovir.

Supplementary Table S3: Association of socioeconomic status and 25(OH)D_3_ with dietary calcium and vitamin D dietary consumption

|  |  | Dietary Calcium, n (%) | | | Dietary vitamin D, n (%) | | | |
| --- | --- | --- | --- | --- | --- | --- | --- | --- |
|  |  | **<150mg** | **150-299mg** | **≥300mg** | **<4.0μg** | **4.0-5.9μg** | **≥6.0μg** |  |
| SES | Poorest | 146 (85.9) | 19 (11.2) | 5 (2.9) | 47 (27.7) | 107 (62.9) | 16 (9.4) |  |
|  | 2 | 137 (82.0) | 21 (12.6) | 9 (5.4) | 41 (24.6) | 93 (55.7) | 33 (19.8) |  |
|  | 3 | 133 (76.0) | 25 (14.3) | 17 (9.7) | 36 (20.6) | 107 (61.1) | 32 (18.3) |  |
|  | 4 | 117 (72.2) | 26 (16.1) | 19 (11.7) | 21 (13.0) | 106 (65.4) | 35 (21.6) |  |
|  | Richest | 106 (63.1) | 38 (22.6) | 24 (14.3) | 28 (16.7) | 83 (49.4) | 57 (33.9) |  |
| 25(OH)D_3_ | >75 nmol/L | 156 (76.9) | 32 (15.8) | 15 (7.4) | 36 (17.7) | 120 (59.1) | 47 (23.2) |  |
|  | 50-75 nmol/L | 392 (75.4) | 80 (15.4) | 48 (9.2) | 101 (19.4) | 311 (59.8) | 108 (20.8) |  |
|  | <50 nmol/L | 91 (76.5) | 17 (14.3) | 11 (9.2) | 36 (30.3) | 65 (54.6) | 18 (15.1) |  |

SES: socioeconomic status

Supplementary Table S4: Mean and standard deviation of bone density and muscle function outcomes by 25(OH)D_3_ concentrations

| 25(OH)D_3_ | N | TBLH-BMD^HT^ Z-score, mean (SD) | LS-BMAD Z-score, mean (SD) | Grip strength-for-height Z-score, mean (SD) | Jump power-for-height Z-score, mean (SD) |
| --- | --- | --- | --- | --- | --- |
| >75 nmol/L | 203 | -0.87 (0.98) | -0.67 (1.15) | 2.59 (1.22) | -0.18 (1.53) |
| 50-75 nmol/L | 520 | -0.98 (0.98) | -0.78 (1.11) | 2.34 (1.12) | 0.05 (1.52) |
| <50 nmol/L | 119 | -1.31 (1.03) | -0.10 (1.26) | 2.19 (1.05) | 0.11 (1.11) |

TBLH-BMD: total body less head bone mineral density. LS-BMAD: lumbar spine bone mineral apparent density
