# Supplementary figures and images for: Association Between Vitamin D Insufficiency and Impaired Bone Density Among Adolescents With Perinatally Acquired HIV Infection
Source: Open Forum Infect Dis. 2024 Sep 19;11(9):ofae442. doi: 10.1093/ofid/ofae442 (PMC11411771; doi:10.1093/ofid/ofae442)

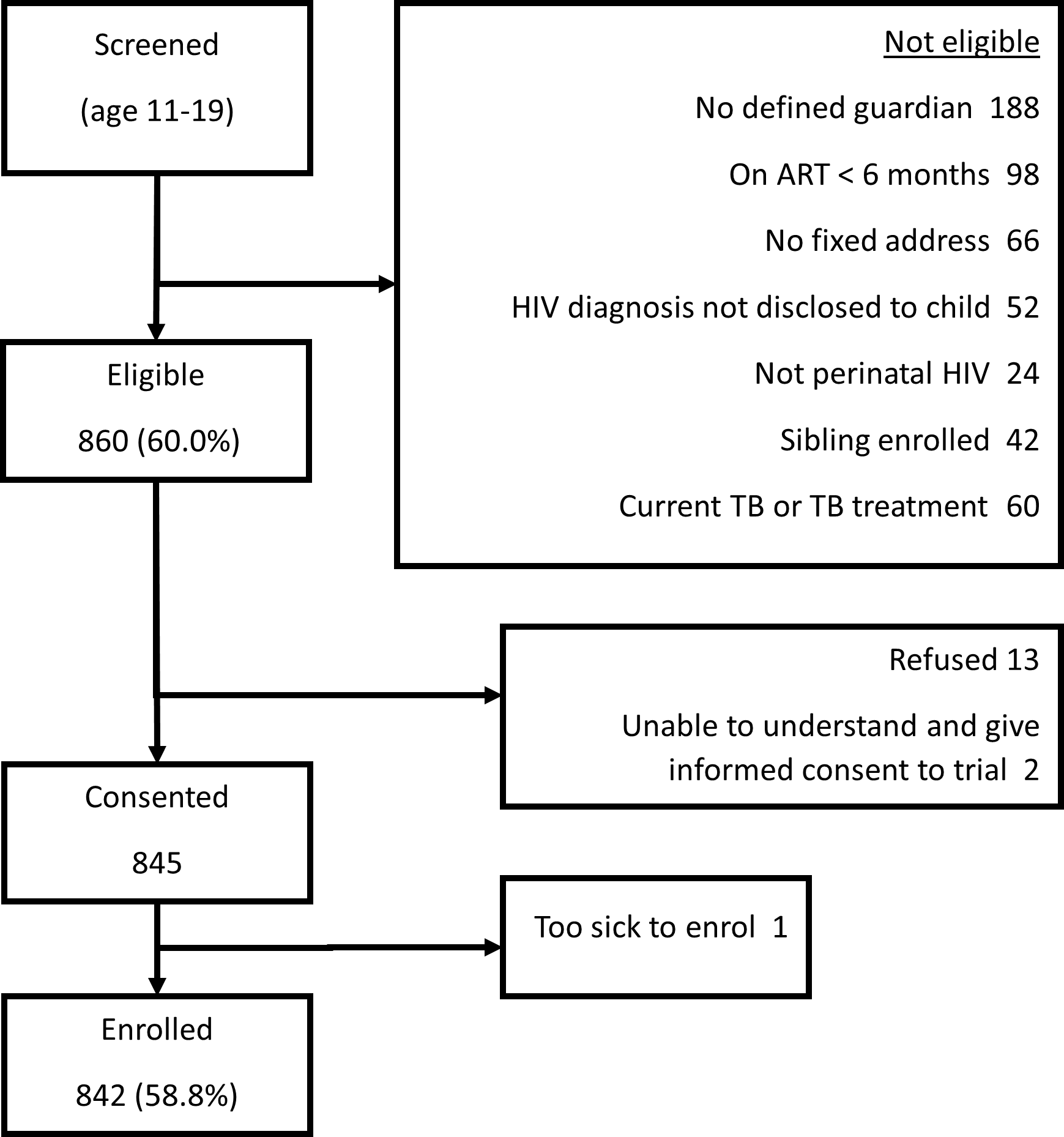

Supplement: ofae442_Supplementary_Data [file ofae442_supplementary_data.zip › Supp Fig 1.tif]
